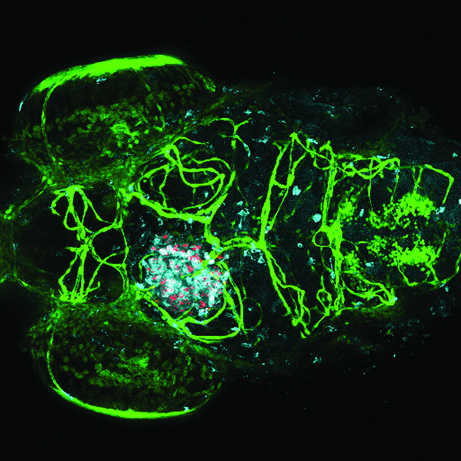# Insights into granuloma formation in tuberculous meningitis using the zebrafish-*M. marinum* model

**Published:** 2014-09

*Mycobacterium tuberculosis* typically affects the lungs, causing tuberculosis, but can occasionally spread to the CNS via the bloodstream, leading to tuberculous meningitis (TBM), a life-threatening complication. A key pathological feature of TBM is the formation of granulomas – collections of immune cells – in the brain or meninges (connective tissue layers that envelop the CNS). Little is known about the role of granulomas in the early phases of TBM development. Astrid van der Sar and colleagues sought to explore the pathogenesis of CNS granuloma formation at different developmental stages, using a well-established zebrafish model of tuberculosis in which infection is caused by *Mycobacterium marinum*, a close relative of *M. tuberculosis*. The authors first confirmed that *M. marinum* infection induces TBM in adult zebrafish and then looked at earlier stages of development. To determine the optimum mode of inoculation in zebrafish embryos, they used local and systemic inoculation routes; infection via the bloodstream or directly into the CNS resulted in the formation of CNS granulomas. Interestingly, granulomas formed regardless of whether infection occurred before or after development of the blood-brain barrier, indicating that the pathogen can efficiently cross this barrier. Inactivation of the mycobacterial ESX-1 virulence secretion system, which is important for pathogenesis, resulted in the formation of smaller clusters with a high number of phagocytosed bacteria. However, migration of bacteria from the bloodstream to the brain did not seem to be affected, suggesting that the ESX-1 locus is dispensable for migration. This study shows that the zebrafish-*M. marinum* model provides an effective tool for characterisation of host-pathogen interactions in granuloma formation. This model could be used to explore the pathogenesis of TBM and pinpoint potential markers for early disease diagnosis and treatment. **Page 1111**

**Figure:**